# Older age is a protective factor for academic achievements irrespective of treatment modalities for posterior fossa brain tumours in children

**DOI:** 10.1371/journal.pone.0243998

**Published:** 2020-12-16

**Authors:** Jarmila Kruseova, Anna Sarah Kovacova, Michal Zapotocky, David Sumerauer, Ivana Pernikova, Darja Starkova, Adela Misove, Andrea Zichova, Vaclav Capek, Thorsten Langer, Antoinette am Zehnhoff-Dinnesen, Tomas Eckschlager, Martin Kyncl

**Affiliations:** 1 Department of Paediatric Haematology and Oncology, Charles University, 2^nd^ Faculty of Medicine and University Hospital Motol, Prague, Czech Republic; 2 Department of Radiology, Charles University, 2^nd^ Faculty of Medicine and University Hospital Motol, Prague, Czech Republic; 3 Department of Paediatric Neurology, Charles University, 2^nd^ Faculty of Medicine and University Hospital Motol, Prague, Czech Republic; 4 Bioinformatics Centre, Charles University, 2^nd^ Faculty of Medicine and University Hospital Motol, Prague, Czech Republic; 5 Pediatric Oncology and Hematology, University Hospital for Children and Adolescents, Lübeck, Germany; 6 Department of Phoniatrics and Pedaudiology, University Hospital Münster, Münster, Germany; Universidade Federal de Sao Paulo/Escola Paulista de Medicina (Unifesp/epm), BRAZIL

## Abstract

The treatment of children with posterior fossa brain tumours (PFBT) impacts their long term functional and imaging outcomes. This study aimed to evaluate academic achievement correlated with long-term sequelae after different PFBT treatment modalities. The study cohort consisted of 110 survivors (median age at diagnosis 10.1 years and median time of follow up 13.2 years) who completed hearing questionnaires, neurological assessment and MRI of the brain ≥5 years after the end of treatment. There were three treatment groups. A cisplatin group which underwent cisplatin chemotherapy, radiotherapy and surgery (medulloblastoma N = 40), a radiotherapy group which underwent radiotherapy and surgery (astrocytoma/ependymoma N = 30), and a surgery group (astrocytoma N = 40). Academic achievement was correlated to the age at diagnosis, ototoxicity, Karnofsky score (KS), and MRI findings (Fazekas Score (FS)- treatment related parenchymal changes). For a modelled age at diagnosis of five years, the cisplatin group had lower academic achievements compared to the radiotherapy (p = 0.028) and surgery (p = 0.014) groups. Academic achievements evaluated at a modelled age of 10 years at diagnosis did not significantly differ among the treatment groups. The cisplatin group exhibited a higher occurrence of ototoxicity than the radiotherapy (p<0.019) and surgery groups (p<0.001); however, there was no correlation between ototoxicity and academic achievements (p = 0.722) in older age at diagnosis. The radiotherapy group exhibited lower KS than the surgery group (p<0.001). KS significantly influenced academic achievements in all groups (p<0.000). The cisplatin group exhibited higher FS than the surgery group (p<0.001) while FS did not correlate with academic achievement (p = 0.399). Older age is a protective factor for academic achievements irrespective of a treatment modality.

## Introduction

With the advent of modern treatment approaches, more than 70% of children diagnosed with primary Central Nervous System (CNS) cancers are surviving longer than five years after the end of treatment [1–3]. One of the factors contributing to this increased number of survivors is the result of intensified therapies, many of which employ cisplatin-based regimens and radiotherapy. However, long-term toxicity remains a major problem for these survivors which significantly affects their quality of life [1–3]. Sensorineural hearing loss (SNHL) is an important consequence of cisplatin chemotherapy [4–6]. Cranial radiotherapy is less ototoxic than cisplatin-based treatment but is still associated with a high risk of SNHL which is even more pronounced when combined with cisplatin [1, 5]. Though studies confirmed a correlation between hearing loss and academic achievements in patients younger than 5 years at diagnosis [1,5], longitudinal reports of SNHL in childhood cancer survivors are limited [4, 6]. Chemo-irradiation and tumour-related effects lead to other long-term consequences, including neurocognitive deficits such as learning and memory deficits [1, 4]. This can negatively influence academic achievements [3, 4, 7, 8]. Posterior fossa brain tumour (PFBT) survivors can be affected by all of the late neurological effects mentioned above [7, 9]. However, the relative role of different neurotoxic effects of PFBT treatment modalities are poorly understood [7]. This central neurotoxicity may manifest as radiological imaging abnormalities [7–10]. The most studied are these two changes the alteration of white matter (WM) and cerebral atrophy (CA) which are detectable by MRI [7–10]. These MRI findings develop more frequently after chemo-irradiation compared to radiotherapy alone and are associated with poor cognitive performance and IQ scores [9, 10]. To our knowledge, there has been no published study of PFBT survivors and correlations between academic achievement, MRI findings and late effect sequelae with a median follow-up longer than 12 years.

In the current study we took advantage of a long follow-up of our PFBT survivors to clarify the role of older age at diagnosis in cases of different treatment modalities. Firstly, we aimed to evaluate academic achievement correlated with age at diagnosis and long-term sequelae after PFBT, using different treatment modalities. Secondly, we correlated the presence of these late effects among PFBT survivors and MRI brain imaging findings.

## Material and methods

### Patient cohort

We conducted a retrospective study of long-term survivors treated for PFBT at the Department of Paediatric Haematology and Oncology, University Hospital Motol, Prague, Czech Republic, between the years 1980–2012. Within this period, the total number of CNS tumour survivors were 634. Survivors treated with methotrexate (neurotoxicity risk) (N = 31), who had genetic syndromes (N = 98) or developed relapses (N = 52) or subsequent neoplasms (N 32) were excluded. We excluded patients who reported hearing impairments in their family history, including siblings (N = 3). From the remaining cohort 110 of whom were included subject to the application of the following inclusion criteria: 1) PFBT survivors; 2) completed hearing questionnaire as part of the PanCareLIFE* ototoxicity study (2014–2017); 3) neurological assessment; and 4) MRI of the brain (2014–2017). All correlations were conducted using data collected at least five years after the end of treatment.

We evaluated academic achievement in PFBT survivors with regard to age at diagnosis and other long-term consequences, such as ototoxicity, Karnofsky Score (KS), and MRI findings. PFBT survivors were divided into three groups according to the therapy they had received: the cisplatin group received all treatment modalities [cisplatin-based regimens, radiotherapy, and surgery] (N = 40); the radiotherapy group consisted of patients who had undergone radiotherapy and surgery (N = 30); and the surgery group comprised patients who had undergone a surgical procedure only (N = 40). The observed groups did not differ statistically in age at diagnosis, sex, and age during evaluation. The presence of decompensated hydrocephalus at diagnosis with ventriculoperitoneal (VP) shunt placement during study evaluation and follow-up was higher in the radiotherapy group. The patients’ characteristics are summarized in Table 1.

**Table 1:** Patients characteristics.

Characteristics	Cisplatin group	Radiotherapy group	Surgery group	Significance among groups
Total number of patiens	N 40	N 30	N 40	
Female/ Male	**17(43%) / 23 (57%)**	**18 (60%) / 12 (40%)**	**18(45%)/22 (55%)**	**p = 0.308**
Histopathology	Medulloblastoma	Ependymoma gr.III 9 (30%)	Astrocytoma gr. I 26(65%)	--
	Classic 15(38%)			
	Desmoplastic 6(15%)	Astrocytoma gr. II/III 17 (57%)	Astrocytoma gr.II 14(35%)	
	Anaplastic 5(12%)			
	Unclassified 14(35%)	Astrocytom gr. I 4 (13%)		
Hydrocephalus at dg. ^1^+ VP shunt ^2^ number of patients.	N7 (17%)	N 13 (43%)	N 10 (25%)	p = 0.052
Median ± IQR^●^ age at dg. ^2^ (years)	9.5 (6.7–11.9)	12.4 (5.8–17.7)	11.1 (5.6–14.3)	p = 0.366
Median ± IQR^●^ FUP^3^ since dg. ^1^ (years)	12.4 (8.6–15.5)	14.7 (11.8–17.5)	11.9 (9.2–14.6)	P = 0.050*
Median ± IQR^●^ age at the time of investigation (years)	22.3 (19.5–27.1)	24.5 (21.2–32.2)	22.5(18.0–25.9)	p = 0.080
Treatment period	1990–1999 N7 (18%)	1991–1999 N9 (30%)	1992–1999 N6(15%)	--
Years/number of patients	2000–2004 N17 (42%)	2000–2004 N15 (50%)	2000–2004 N14(35%)	
	2005–2011 N16 (40%)	2005–2011 N 6 (20%)	2005–2012 N20(50%)	
Subtotal/Complete surgery	13 (33%)/ 27(67%)	18 (60%)/ 12 (40%)	13(33%)/27(67%)	--
Radiotherapy			--	--
Posterior fossa dose	55.8 Gy (50.0–59.8)	54.6Gy (50–59.8)		
Craniospinal dose	25.4 Gy (24.9–30.6)	N2 30 Gy		
Cisplatin median dose	470.5 mg/m^2^	--	--	--
CCGA9961^4^				
Standard risk patients N = 25	544 mg/m^2 6^			
High risk patients N = 11	300 mg/m^2 7^			
7 in one protocol^5^ N = 4	480 mg/m^2 8^			

* statistically signifiant, ^●^ IQR: interquartile range, ^1^ dg.–diagnosis, ^2^ hydrocephalus and VP shunt–initially decompensated hydrocephalus and than ventriculoperitoneal shunt placement, ^3^ FUP- follow up.

^4–5^ Medulloblastoma treatment protocols with cisplatin: CCGA9961—Children's Cancer Group/Pediatric Oncology Group study number A 9961 and "7 in one protocol". Study patients received following cumulative dose of cytostatics according these protocols.

CDDP—cisplatin, VCR—vincristine, CCNU—lomustine, CYC–cyclophosphamide.

^6^CDDP 544 mg/m^2^–8 courses 68 mg/m^2^, concomitant cytostatic: VCR 48 mg/m^2^, CCNU 600 mg/m^2.^.

^7^ CDDP 300 mg/m^2^–4 courses 75 mg/m^2^, concomitant cytostatics: VCR 12 mg/m^2^, CYC 16000 mg/m^2^.^.^

^8^ CDDP 480 mg/m^2^–6 courses 80 mg/m^2^, concomitant cytostatics: VCR 12 mg/m^2^, CYC 2400 mg/m^2^, CCNU 600 mg/m^2^, procarbazine 600 mg/m^2^, cytarabine 2400 mg/m^2^, hydroxyurea 12 000 mg/m^2.^.

### Academic achievements

We correlated academic achievements for patients > 22 years (N = 85) (Scale: 0 psychomotor retardation, 1 primary school, 2 secondary school without graduation, 3 high school, 4 university).

### Hearing impairment evaluation

As part of the PanCareLIFE* study (2014–2017), all participants filled out self-reported questionnaires regarding hearing impairment, hearing aids, and tinnitus. For statistical evaluation, we used the following scale: 0 = no hearing impairment; 1 = self-reported hearing problems including tinnitus without the need of a hearing aid; 2 = hearing aid; and 3 = deafness. The definition of severe ototoxicity in present study was grade 2 and grade 3 together. All patients had normal hearing before oncology treatment.

### Neurological outcomes

For neurology assessment we used standard KS evaluation [11] and incidence of epilepsy based on the need for current medication. The neurological examination included evaluation of: 1) motoric functions (muscle tone, tendon reflexes, cerebellar functions, fine hand motor skills; 2) neurocognitive functions (attention, memory, processing speed); 3) practical skills and 4) mental functions (conclusions of school reports, the need for certain adjustments, individual programs, emotional and adjustment problems, working experiences, questions about other activities and hobbies). KS definition: 100 = normal, no complaints; 90 = able to carry on normal activities, with minor signs or symptoms of disease; 80 = normal activity with effort, with some signs and symptoms of disease; 70 = cares for self but unable to carry on normal activity or to perform work; 60 = requires occasional assistance but is able to take care of most personal needs; 50 = requires frequent assistance and medical care; 40 = disabled, requires special care and assistance; and 30 = severely disabled. All of our study’s long-term survivors were seen by one senior neurologist specializing in cancer late effects.

### Imaging outcome measures

MRI scans were retrospectively reviewed for the following changes: brain atrophy, surgery-related focal abnormalities, and chemo/radiotherapy treatment-related focal or generalized parenchymal changes. Brain atrophy was assessed using a subjective visual grading system categorized as absent or present. Postoperative parenchymal changes were recorded as appropriate or extended by subjective visual grading. As part of the categorization of focal brain lesions, we used the Fazekas score (FS) to detect T2 / FLAIR hyperintense foci. FS was categorized according to the treatment-related parenchymal changes (0 none/single lesion; 1 multiple punctate; 2 beginning confluence of lesions; 3 large confluent lesions). When employing FS, we used the following methodologies [12, 13]. The MRI studies included FLAIR sequences (slice thickness 4–5 mm). Each MR image was evaluated independently by two readers in consensus: a senior consultant radiologist with expertise in paediatric radiology and a resident radiologist with 4 years of experience in paediatric imaging in the field. The radiologists did not have information about detailed oncology treatment and subsequent complications. MRI was conducted on 1.5 Tesla scanners (Achieva and Intera, Philips, The Netherlands, Avanto, Siemens, Germany).

### Statistical analysis

Differences in baseline Patients´ characteristics among the studied groups of patients, see Table 1, were studied using chi^2^ test, parametric ANOVA and the Kruskal-Wallis test. Risks of ototoxicity, epilepsy, tinnitus, atrophy, post-operative changes, and FS were modelled by logistic regression. Influence of the treatment and the age at diagnosis on the highest education level was examined using a multivariable ordinal logistic regression with an interaction between the treatment and the age. In this model possible confounders (gender, FUP, and hydrocephalus with VP shunt placement) were also considered. Effect of the treatment on KS was modelled by linear regression with a dependent variable being transformed using a Box-Cox transformation. All p values are reported as two-sided, using 0.05 as the level of significance. Analyses were conducted using an R statistical package, version 3.4.2, R Core Team (2017).

### Ethics statement

Written informed consent for using their ototoxicity and education questionnaires was obtained from all study participants > 18 years and for participants ˂ 18 years was obtained from parents/legal guardians for these patients. Those results were part of PanCareLife Studies in Fertility and Ototoxicity to Improve Quality of Life after Cancer during Childhood, Adolescence and Young Adulthood—we received ethical favorable opinion–Ethics Committee for Multi-Centric Clinical Trials of the University Hospital Motol 1.4.2014 –Reference No.: EK-478/14. Neurology assessment and brain MRI were part of standard routine follow up care–all included patients provided informed written consent to have data from their medical records used in research with strictly anonymous data. Ethical approval of this part of present study was waived by the local Ethics Committee of Motol University Hospital in view of the retrospective nature of the study and all the procedures being performed were part of the routine care. We retrospectively evaluated medical records 4.2014–12.2017.

## Results

### Age at diagnosis strongly influences academic achievements in PFBT survivors

The modelled age of 5 years at diagnosis for the cisplatin group exhibited significantly decreased academic achievements compared to the radiotherapy and surgery groups. In contrast, academic achievements evaluated at a modelled age of 10 years at diagnosis did not significantly differ among the groups. We confirmed a negative young age effect at diagnosis on further academic achievements only in the cisplatin group (p = 0.003). From 85 patients > 22 years six patients completed university studies (15%) in the cisplatin group, compared to 9 patients (30%) in the radiotherapy group and 9 patients (23%) in the surgery group. Statistical correlations of academic achievements among the observed groups and the modelled age at diagnosis are shown in Table 2.

**Table 2:** Academic achievements–multivariable model.

Parameter	Estimate	SE	z-value	p-value
**A. Full model containing all confounders considered**				
surgery group^1^	4.6495	1.0570	4.3986	0.0000
radiotherapy group^2^	4.4431	1.1959	3.7152	0.0002
Age	0.3892	0.0838	4.6441	0.0000
FUP	0.1011	0.0374	2.7010	0.0069
Hydrocephalus at dg.+ VP shunt	0.8430	0.4319	1.9518	0.0510
Gender	0.1066	0.3750	0.2844	0.7761
surgery group:age	-0.3675	0.0997	-3.6842	0.0002
radiotherapy group:age	-0.4311	0.1151	-3.7451	0.0002
**B. Reduced model containing only statistically significant parameters**				
surgery group	4.2479	1.0347	4.1053	0.0000
radiotherapy group	3.9745	1.1584	3.4310	0.0006
Age	0.3800	0.0826	4.6011	0.0000
FUP	0.1018	0.0365	2.7880	0.0053
surgery group:age	-0.3360	0.0977	-3.4381	0.0006
radiotherapy group:age	-0.4079	0.1125	-3.6248	0.0003
**C. Post-hoc tests of the effect of age in treatment groups**				
cisplatin group	0.380	0.083	4.601	0.000
surgery group	0.044	0.059	0.744	0.839
radiotherapy group	-0.028	0.074	-0.379	0.974
**D. Post-hoc tests of the difference between treatment groups in modelled age of 5 years**				
cisplatin vs. surgery group	2.568	0.620	4.142	0.000
cisplatin vs. radiotherapy group	1.935	0.687	2.817	0.013
surgery vs. radiotherapy group	-0.633	0.656	-0.965	0.598
**E. Post-hoc tests of the difference between treatment groups in modelled age of 10 years**				
cisplatin vs. surgery group	0.888	0.419	2.119	0.086
cisplatin vs. radiotherapy group	-0.105	0.485	-0.216	0.975
surgery vs. radiotherapy group	-0.992	0.488	-2.032	0.104

^1^surgery group: age–interaction between age and the effect of surgery with respect to cisplatin.

^2^radiotherapy group: age–interaction between age and the effect of radiotherapy with respect to cisplatin.

### Significant risk of hearing impairment after radiation and cisplatin-based therapy

The overall incidence of self-reported hearing problems in the observed groups was 25%. The cisplatin group had 21 patients (53%) who reported hearing impairment, including eight patients (21%) with severe ototoxicity (hearing aid in seven patients and deafness in one patient). The radiotherapy group had six patients (20%) with four suffering from severe ototoxicity (13%) (hearing aid in three patients and deafness in one patient). In the surgery group, only one patient had a mild hearing impairment. Statistical correlations of hearing impairments among the observed groups are provided in Table 3. We did not confirm any correlation between hearing impairment and academic achievements (p = 0.723).

**Table 3:** Hearing impairment.

Risk factors	Estimate	SE	z- value	p-value
**Hearing impairment**				
Cisplatin group vs. surgery gr.	3.764	1.061	3.547	0.001**
Cisplatin group vs. radiotherapy gr.	1.486	0.556	2.676	0.019*
Surgery gr. vs. radiotherapy gr.	-2.277	1.111	-2.050	0.094

### Performance status scores depend on treatment modalities and the presence of hydrocephalus

**Fig 1:**
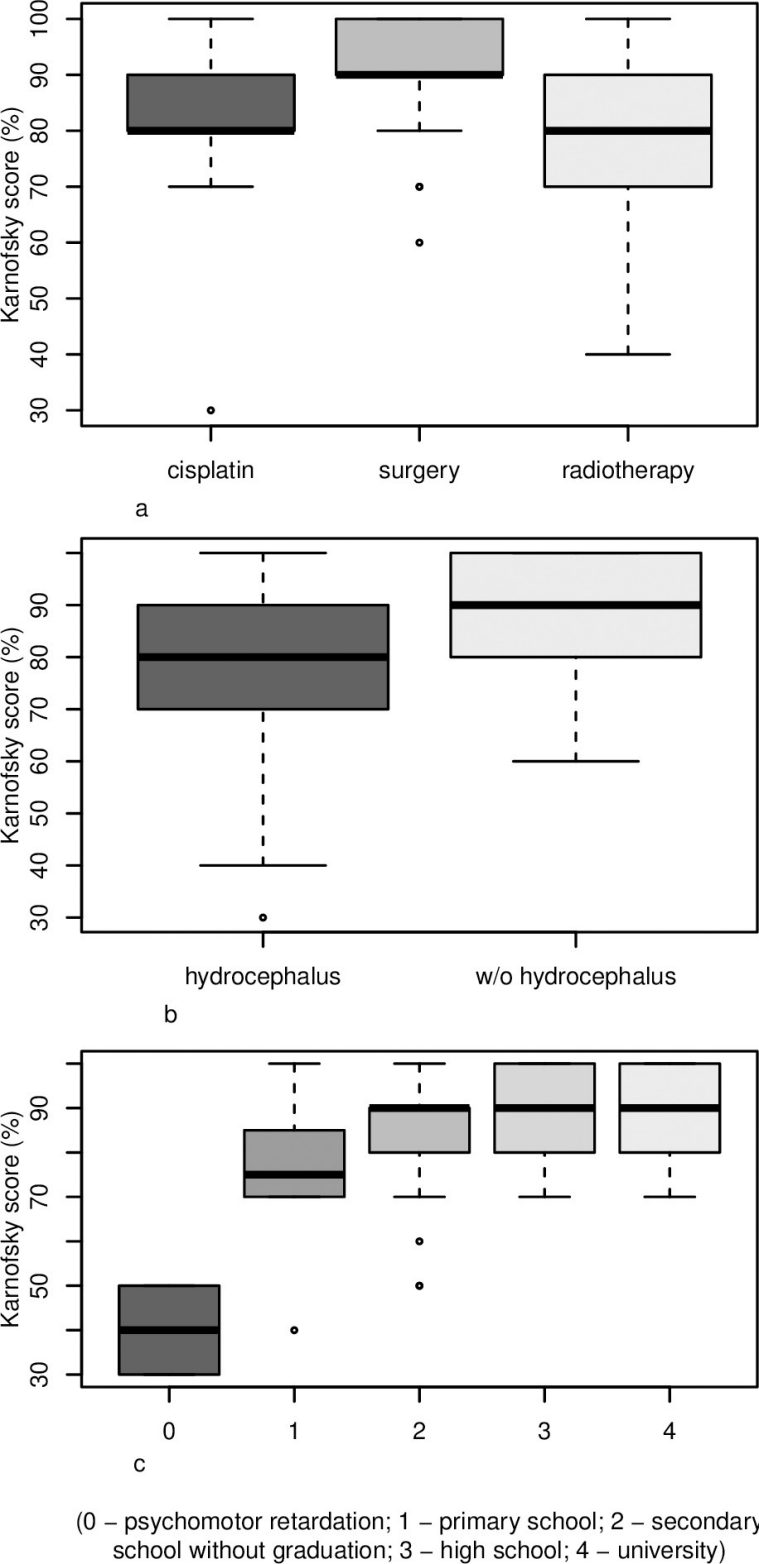
**A.** Comparison of the treatment groups and KS score distribution. **B.** Comparison of hydrocephalus diagnosed at cancer diagnosis and its effect on KS distribution. **C.** Comparison of KS score distribution and its effect on academic achievements.

KS scores of 90–100 were observed in 17 patients (43%) in the cisplatin group, in 11 patients (36%) in the radiotherapy group, and in 33 patients (83%) in the surgery group. For KS distribution among the observed groups see Fig 1A. KS was significantly lower in the cisplatin group vs. surgery group (p˂0.010) as well as in the radiotherapy group vs. surgery group (p˂0.001), but no significant difference was observed between the cisplatin and radiotherapy groups (p = 0.628). We confirmed correlation between decreased KS and the presence of decompensated hydrocephalus at dg. and VP shunt placement in all observed groups (p = 0.022) Fig 1B. There was no correlation between academic achievement and the presence of hydrocephalus at diagnosis (p = 0.110). Highly significant correlation between lower KS and worse academic achievement (p˂0.000) was confirmed in all groups Fig 1C.

### Treatment-related MRI changes correlate with performance status

**Fig 2:**
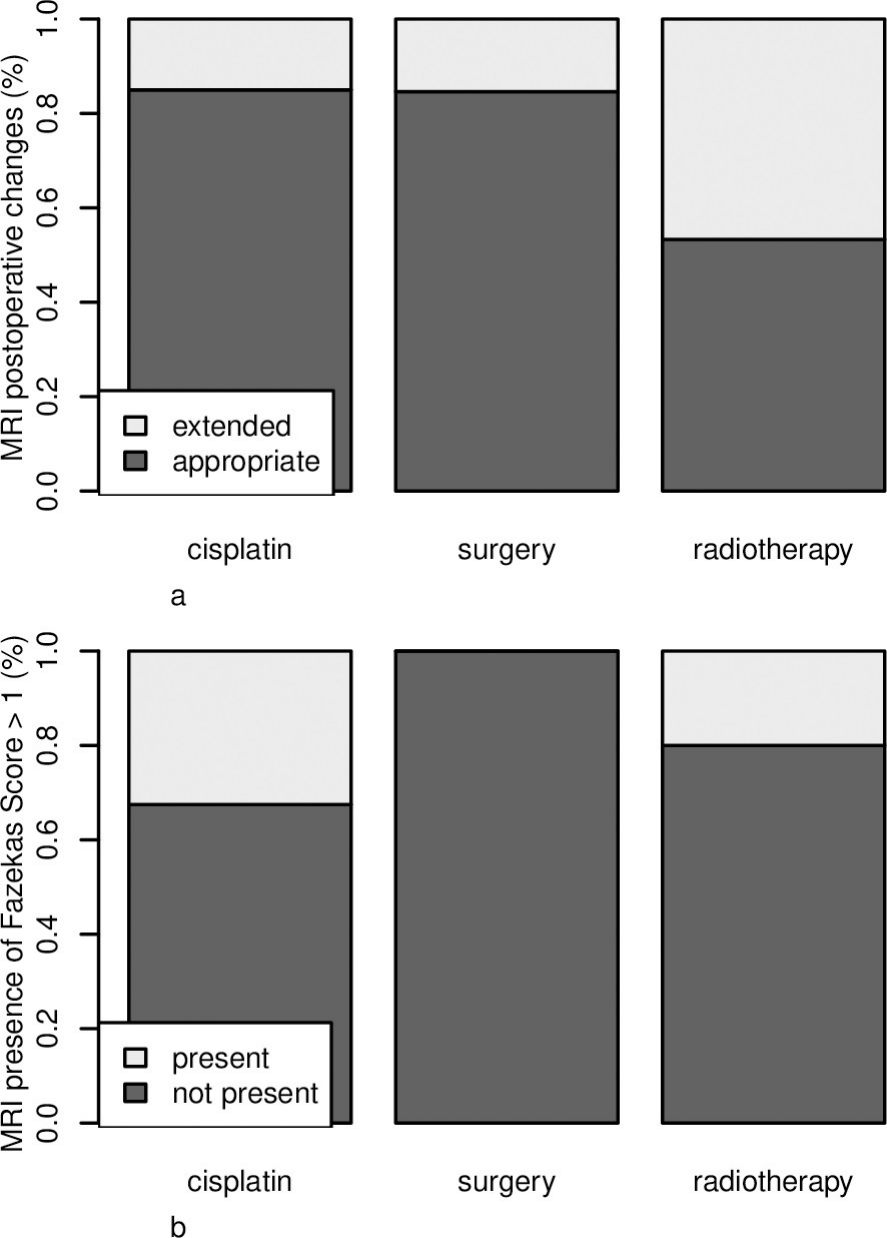
**A.** Comparison of the treatment groups and MRI presence of postoperative changes. **B.** Comparison of the treatment groups and MRI presence of FS>1.

Brain atrophy was observed in 11 patients (27%) in the cisplatin group, in four patients (13%) in the radiotherapy group, and in eight patients (20%) in the surgery group. We did not observe any group effect on brain atrophy development (p = 0.488). Extensive postoperative changes were seen in six patients (15%) in the cisplatin group, in 14 patients (47%) in the radiotherapy group, and in seven patients (18%) in the surgery group Fig 2A). There were differences between the radiotherapy group and the cisplatin group (p˂0.014) and the surgery group (p˂0.017). We did not confirm correlation between the extent of postoperative changes and academic achievements (p = 0.35). White matter changes characterized by FS >1 were seen in 13 patients (33%) in the cisplatin group and in six patients (20%) in the radiotherapy group (p = 0.189). The surgery group had no WM changes Fig 2B. Analysis confirmed that higher FS correlated with worse KS in all groups (p = 0.033). Furthermore, young age at diagnosis correlated with more extensive white matter changes and higher FS was observed (p˂0.009). Correlation of FS and academic achievement was on the border of statistical significance in the cisplatin group (p = 0.072). When evaluating all groups together, there was no correlation between FS and academic achievements (p = 0.399).

## Discussion

One of the key questions for cancer survivors who are cured is “what my quality of my future life will be“. The level of academic achievement is of great importance for many survivors. Our study provides and explains which treatment modalities and late sequelae affect academic achievements. Compared to previously published studies [4, 5], our analysis was based on different treatment modalities in long-term PFBT survivors who were older at diagnosis.

The overall incidence of self-reported hearing problems in our groups was 25%. These findings are similar to previous Children Cancer Group (CCG) study where the prevalence of self-reported hearing loss was 20% [14]. Severe ototoxicity was present in 35% of patients in the cisplatin group compared to 20% of patients in the radiotherapy group, what is in accordance with previous ototoxicity studies [1, 15, 16]. We did not identify correlation between self-reported hearing impairments and academic achievements, which is in contrast to Brinkman et al [1] who observed that hearing loss was perceived to have a negative impact on education. In their study, the prevalence of serious ototoxicity was 36%. Although our cisplatin group had the same prevalence of serious hearing loss (35%) we did not confirm their results. A possible explanation follows from the fact that our study included children who were older at the time of diagnosis. Previous studies reported the effects of hearing loss with regards to academic achievement but all in patients younger than 5 years at diagnosis [1, 5, 16].

We observed a negative effect of young age at diagnosis on further academic achievements only in the cisplatin group. This finding is in conformity with a theory regarding CNS survivors having a reduced ability to learn new information with a significant effect of age at diagnosis [2]. We confirmed that older age at diagnosis did not affect academic achievements among all of the observed groups despite the treatment modality applied. Therefore, this result and our previous findings further support our hypothesis of the “protective effect of age”.

Neurotoxicity due to specific chemotherapeutic agents has been described for methotrexate, alkylating agents (i.e., cisplatin, ifosfamide, cyclophosphamide) and others such as carmustine, cytosine arabinoside, and vincristine [8, 9, 17–19]. In order to better understand the impact of cisplatin on academic achievements, we excluded all regimens comprising methotrexate which quite often results in learning disabilities [8, 17, 19]. Our patients were treated with chemotherapeutic regimens that included some of the cytostatics mentioned above; therefore, there might be an additive effect of other cytostatics on cisplatin itself. We found the highest difference in academic achievements in younger children between medulloblastoma and pilocytic astrocytoma, which is in line with the study of Duffner et al [17] who found that children with medulloblastoma had significantly worse IQ than children with cerebellar astrocytoma.

PFBT survivors can develop a range of persistent late effects in neuro-cognitive, sensation, and functional abilities [3, 9–11, 20–22]. We decided to use KS because it is the most complex available tool for neurology assessment. There was a strong correlation between KS and academic achievements irrespective of treatment modalities. We did not observe additional negative effects of cisplatin treatment on KS. No worsening of KS was present even in case of whole craniospinal irradiation in the cisplatin group compared to focal PFBT irradiation in the radiotherapy group. In concordance with the work of Rueckriegel et al. [7], our study revealed that hydrocephalus at diagnosis also contributed to a decrease in KS. In contrast to a study by Lassaletta et al. [22], in which children with PFBT and the presence of hydrocephalus had significantly worse academic outcomes, we did not observe this correlation. Late effects tend to be aggravated with time [3–5, 23]; consequently, we suppose that another factor contributing to the lowest KS in the radiotherapy group could be the longer follow-up time in this group.

Conventional magnetic resonance imaging (MRI) can identify some long-term changes of the central nervous tissue, such as morphologic local damage, brain atrophy, and alterations of WM [7, 9, 12, 24–28]. The highest number of WM changes were seen in the cisplatin group, which is consistent with previous publications where medulloblastoma treatment exhibited a high risk of WM damage [7, 9, 21]. The differences in FS between the cisplatin and radiotherapy groups did not reach statistical significance, but we supposed that there could be some additive effects of cisplatin-based regimens on WM changes. Unlike previous studies in which MRI abnormal WM volume correlated with increasing deficits in intelligence and academic performance [9, 24], we could not adamantly support this finding in our study cohort. We only confirmed correlation between FS and KS in the cisplatin and radiotherapy groups. It was only FS that showed the group effect of age at diagnosis. Younger patients at the beginning of treatment had higher FS [9]. Since we included older patients at diagnosis, we assumed that FS was not so frequent in our study cohort and we did not observe negative influence of WM changes on academic achievement.

The limitations of our study are the longer follow-up and the higher presence of hydrocephalus at diagnosis in the radiotherapy only patients, which could influence the evaluation of the additional effects of the cisplatin treatment. Another limit of this study aimed at assessing educational attainment is that the presence of various other chronic consequences from previous cancer treatment (endocrine, renal, cardiac, sleeping disorders, chronic fatigue syndrome etc.) and survivors psychological well-being may influence academic achievement. The family, social conditions, and motivation also play an important role. These factors were not in current study evaluated.

## Conclusions

Our long-term data were able to compare different late sequelae in patients treated for PFBT. We can confirm that the group of younger children who were treated with surgery, craniospinal radiation, and cisplatin-based regimen were the most vulnerable one, having the highest incidence of hearing impairments and the lowest academic achievement. Treatment modalities in all groups did not differ in their effects on academic achievement among older children. We did not observe, except KS, any impact of ototoxicity and MRI findings after different PFBT treatments on academic achievements in patients who were older at diagnosis.

## Supporting information

S1 Dataset List of patients data and outcomes. (XLSX)
